# Age and outcomes in people with type 2 diabetes admitted to hospital with COVID‐19: A cohort study

**DOI:** 10.1111/dme.15481

**Published:** 2024-11-19

**Authors:** M. Ni'Man, Y. Ruan, J. Davies, S. Harris, D. Nagi, P. Narendran, B. C. T. Field, I. Idris, D. Patel, R. Rea, R. E. J. Ryder, S. H. Wild, K. A. Várnai, E. G. Wilmot, K. Khunti

**Affiliations:** ^1^ Nottingham University Hospitals Nottingham UK; ^2^ University of Nottingham Nottingham UK; ^3^ Oxford Centre for Diabetes, Endocrinology and Metabolism Oxford University Hospitals NHS Foundation Trust Oxford UK; ^4^ Institute of Biomedical and Health Engineering, Shenzhen Institute of Advanced Technology Chinese Academy of Sciences Shenzhen China; ^5^ Oxford NIHR Biomedical Research Centre Oxford UK; ^6^ Diabetes Department King's College Hospital London UK; ^7^ Mid Yorkshire Hospitals NHS Trust Pinderfields Hospital Wakefield UK; ^8^ Diabetes Centre, The Queen Elizabeth Hospital University Hospitals Birmingham NHS Foundation Trust Birmingham UK; ^9^ Faculty of Health & Medical Sciences University of Surrey Guildford UK; ^10^ Department of Diabetes & Endocrinology Surrey & Sussex Healthcare NHS Trust Surrey UK; ^11^ Diabetes Department University Hospitals of Derby and Burton NHS Foundation Trust Derby UK; ^12^ Department of Medicine, University College London Royal Free Campus London UK; ^13^ Sandwell and West Birmingham Hospitals NHS Trust Birmingham UK; ^14^ Usher Institute University of Edinburgh Edinburgh UK; ^15^ Diabetes Research Centre, University Hospitals of Leicester NHS Trust Leicester General Hospital Leicester UK

## 1 INTRODUCTION

Type 2 diabetes has been associated with an increased risk of COVID‐19 severity and mortality.^1^ The incidence of young onset (aged <50 years) T2D (YOT2D) has increased in many countries over the past 30 years, particularly in ethnic minority groups.^2^ Accumulating evidence confirms that individuals with YOT2D have greater risks of developing micro‐ and macrovascular complication.^2^ The reasons for poorer outcomes in YOT2D are not known but associated risk factors include female sex, obesity, low birthweight, family history of T2D and non‐white ethnicity.^2^ In view of the high‐risk phenotype, YOT2D may have worse outcomes following COVID‐19.

The aim of this study was, therefore, to assess outcomes, by age on admission to hospital, in two cohorts with T2D: young (<50 years) and older (≥50 years).

## 2 METHODS

Data for this retrospective cohort study were collected through a nationwide audit between March and December 2020, conducted by the Association of British Clinical Diabetologists (ABCD), of COVID‐19‐related admissions in people with diabetes. Full details of data collection have been published previously.^3^

Whilst there is variance in the definition of YOT2D between studies,^4^ we opted to define the age range of the younger people with type 2 diabetes as <50 years, opposed to the typical <40 years definition for YOT2D. This enabled us to study a larger cohort, whilst still referencing this <40 years cohort.

Continuous data variables were summarised as mean (standard deviation) or median [interquartile range] for normally and non‐normally distributed data, respectively. We compared the characteristics of patients using t‐tests or chi‐squared tests for continuous or categorical data. All statistical analysis was performed using R, version 3.3. *p* < 0.05 was considered statistically significant.

## 3 RESULTS

Data from 279 YOT2D were compared to data from 3476 individuals aged ≥50 years with Type 2 diabetes on admission (see Table 1). Despite the mean age of 43.3 years in the YOT2D cohort, there was a high prevalence of microvascular (21%) and macrovascular disease (12%) and hypertension (48%). On admission to hospital, the YOT2D had higher median [IQR] blood glucose levels (10.6[7.4,15.3] mmol/l, *p* < 0.01) compared to the population with T2D that was older (≥50 years) on admission (9.0[6.7,13.0] mmol/L, *p* < 0.01) and higher mean (SD) BMI (33.7 (9.5) kg/m^2^ vs. 29.1(7.2) kg/m^2^, *p* < 0.01). Larger proportions of YOT2D were on metformin (59% vs. 49%, *p* < 0.01), SGLT2 inhibitors (9% vs. 6%, *p* < 0.01), GLP‐1 receptor agonists (6% vs. 3%, *p* < 0.01) and they had higher HbA1c levels (67 (8.3%) versus 61 (7.7%) mmol/mol, *p* < 0.01). Similar proportions of both groups were treated with insulin (38% vs. 36%, *p* < 0.12). Overall, 6% of the younger cohort presented with diabetic ketoacidosis, double that observed in the older group (3%, *p* < 0.01). Mortality during admission for YOT2D was 12% compared to 36% in the older group (*p* < 0.01).

**TABLE 1:** Clinical outcomes for people with T2D <50 years versus ≥50 years at hospital admission with COVID‐19.

Clinical features	Age at hospital admission		*p*‐value for *t*‐test or chi‐squared test
	<50 years (*N* = 279)	≥50 years (*N* = 3476)	
Sex M/total (%)	166/279 (59%)	2139/3475 (62%)	0.23
Mean Age years (SD)	43.3 (6.4)	74 (11)	<0.01
Ethnicity	White 79/279 (28%)	White 2063/3476 (59%)	<0.01
	Asian 81/279 (29%)	Asian 497/3476 (14%)	<0.01
	Black 35/279 (13%)	Black 270/3476 (8%)	<0.01
	Other 28/279 (10%)	Other 88/3476 (3%)	<0.01
	Unknown 56/279 (20%)	Unknown 558/3476 (16%)	NA
Admission features			
DKA on admission	17/279 (6%)	107/3476 (3%)	<0.01
Mean and median (IQR) admission blood glucose (mmol/l)	12.5 (7.4)	11.0 (6.8)	<0.01
	10.6 [7.4,15.3]	9.0 [6.7,13.0]	<0.01
Mean and median (IQR) Creatinine(mcmol/l)	125 (166)	151 (152)	<0.01
	74 [59, 103]	104 [77, 160]	<0.01
Pre‐COVID characteristics			
Smoker	26/129 (20%)	734/1698 (43%)	<0.01
BMI (kg/m^2^)	33.7 (9.5)	29.1 (7.2)	<0.01
HbA1c (mmol/mol)	67 (26)	61 (21)	<0.01
HbA1c (%)	8.3 (2.4)	7.7 (1.9)	<0.01
Microvascular disease (diabetic nephropathy, foot ulcer, retinopathy, peripheral neuropathy)	54/260 (21%)	1396/3174 (44%)	<0.01
Macrovascular disease (peripheral vascular disease, ischaemic heart disease and cerebrovascular disease)	33/271 (12%)	1470/3342 (44%)	<0.01
Co‐morbidities			
Hypertension	120/252 (48%)	2395/3256 (74%)	<0.01
Dementia	2/223 (1%)	489/2855 (17%)	<0.01
Asthma	50/230 (22%)	385/2899 (13%)	<0.01
COPD	4/216 (2%)	468/2769 (17%)	<0.01
Malignant neoplasm	7/234 (3%)	513/2988 (17%)	<0.01
Treatment on admission			
Metformin	144/244 (59%)	1506/3087 (49%)	<0.01
Sulfonylurea	47/238 (20%)	599/3031 (20%)	0.12
DPP‐4	44/241 (18%)	726/3024 (24%)	<0.01
GLP‐1	15/238 (6%)	93/3000 (3%)	<0.01
SGLT‐2	21/228 (9%)	209/2839 (7%)	<0.01
Insulin	86/229 (38%)	1026/2882 (36%)	0.13
Outcomes			
Death	34/278 (12%)	1233/3443 (36%)	<0.01

*Note*: The *p*‐values in the table represent the significance of the difference between the younger and older cohorts. For this analysis, young people with Type 2 Diabetes was defined as <50 years old.

## 4 DISCUSSION

Our data show that despite their young age, YOT2D admitted to hospital with COVID‐19 had a high prevalence of adverse cardio‐metabolic risk factors and diabetes complications, double the prevalence of DKA on admission compared with the older cohort and high mortality given their age. These data support the view that YOT2D represent an extreme phenotype with multiple cardiovascular risk factors leading to the premature development of micro and macrovascular complications. YOT2D are disproportionately from lower socioeconomic backgrounds,^2^ a further independent risk factor for adverse outcomes related to COVID‐19.^5^ The underlying pathogenic factors of YOT2D such as insulin resistance and obesity also play a role in the observed adverse cardio‐metabolic risk profile.

We were not able to determine whether the inflammatory sequelae of COVID‐19 worsen glycaemic control and/or whether the immunosuppressive effects of contribute to the risk of severe COVID‐19 complications. Irrespective of the underlying cause, the mortality in YOT2D observed here was around 30 times higher than that observed from the primary care data analysis in England which reported an annualised mortality rate in people aged under 50 with diabetes of 0.38% in 2019.^6^ Whilst Barron et al. (2020) looked specifically at in‐hospital deaths, our data looked at people with type 2 diabetes that were admitted to hospital with COVID‐19; of which mortality was one outcome. This meant that although our data is not as well powered as Barron et al. (2020), it benefits from being multi‐centred and having a focus on those admitted with both COVID‐19 and type 2 diabetes.

Further limitations of the study include lack of information on the duration of diabetes and our inability to exclude an incorrect classification of type of diabetes among the study population.

## 5 CONCLUSION

YOT2D admitted to hospital with COVID‐19 represent a high‐risk cohort with multiple co‐morbidities. Overall, 6% of YOT2D with COVID‐19 had DKA on admission to hospital and despite younger age, there was a 12% in‐hospital mortality in this UK cohort. These findings add further evidence of the importance of primary and secondary prevention of type 2 diabetes.

## CONFLICT OF INTEREST STATEMENT

KK has acted as a consultant, speaker or received grants for investigator‐initiated studies for Astra Zeneca, Bayer, Novo Nordisk, Sanofi‐Aventis, Servier, Lilly and Merck Sharp & Dohme, Boehringer Ingelheim, Oramed Pharmaceuticals, Pfizer, Roche, Daiichi‐Sankyo, Applied Therapeutics, Embecta and Nestle Health Science. KK was chair of the ethnicity subgroup of the UK Scientific Advisory Group for Emergencies (SAGE) and is a member of SAGE. KK is supported by the National Institute for Health Research (NIHR) Applied Research Collaboration East Midlands (ARC EM) and the NIHR Leicester Biomedical Research Centre (BRC). EGW has received personal fees from Abbott, AstraZeneca, Dexcom, Eli Lilly, Embecta, Insulet, Medtronic, Novo Nordisk, Roche, Sanofi, Sinocare, Ypsomed and research support from Abbott, Embecta, Insulet, Novo Nordisk, Sanofi. DP advisory work: Astra Zeneca, Boehringer Ingelgeim, Eli Lilly, Sanofi. Educational Work, Eli Lilly, NovoNordisk. MN, YR, JD, SH, DN, PN, BCTF, II, RR, REJR, SHW, KAV no CoI.

## Data Availability

The data that support the findings of this study are available from the corresponding author upon reasonable request.
